# The role of RGS12 in tissue repair and human diseases

**DOI:** 10.1016/j.gendis.2024.101480

**Published:** 2024-12-04

**Authors:** Min Jiang, Hongmei Li, Qiong Zhang, Tongtong Xu, Le Huang, Jinghong Zhang, Huiqing Yu, Junhui Zhang

**Affiliations:** a Department of Geriatric Oncology and Department of Palliative Care, Chongqing University Cancer Hospital, Chongqing 400030, China; b Department of Plastic Surgery, State Key Laboratory of Trauma, Burns and Combined Injury, Southwest Hospital, The Third Military Medical University (Army Medical University), Chongqing 400038, China; c Department of Oncology and Southwest Cancer Center, Southwest Hospital, Third Military Medical University (Army Medical University), Chongqing 400038, China; d Institute of Burn Research, State Key Laboratory of Trauma, Burns and Combined Injury, Southwest Hospital, Third Military Medical University (Army Medical University), Chongqing 400038, China; e General Department of Critical Care Medicine, Zhenjiang Traditional Chinese Medicine Hospital, Affiliated Hospital of Nanjing University of Traditional Chinese Medicine, Zhenjiang, Jiangsu 212003, China; f Army 72nd Group Military Hospital, Huzhou, Zhejiang 313000, China; g Chongqing Key Laboratory of Translational Research for Cancer Metastasis and Individualized Treatment, Chongqing University Cancer Hospital, Chongqing 400030, China

**Keywords:** Cancer, Nervous disorders, Osteoporosis, RGS12, Tissue repair

## Abstract

Regulator of G protein signaling 12 (RGS12) belongs to the superfamily of RGS proteins defined by a conserved RGS domain that canonically binds and deactivates heterotrimeric G-proteins. As the largest family member, RGS12 is widely expressed in many cells and tissues. In the past few decades, it has been found that RGS12 affects the activity of various cells in the human body, participates in many physiological and pathological processes, and plays an important role in the pathogenesis of many diseases. Here, we set out to comprehensively review the role of RGS12 in human diseases and its mechanisms, highlighting the possibility of RGS12 as a therapeutic target for the treatment of human diseases.

## Introduction

G-protein-coupled receptors (GPCRs) are widely expressed in human tissues, and GPCR signaling pathways are involved in regulating a variety of systemic physiological processes.^1^ The disorder of the GPCR signaling pathway causes a series of pathophysiological changes and many diseases.^2^ The G protein coupled to GPCR consists of α, β, and γ subunits.^3^^,^^4^ When GPCR is activated by extracellular ligands, guanosine diphosphate (GDP), which binds to the alpha subunit of G protein (Gα), is converted to guanosine triphosphate (GTP).^5^^,^^6^ Then, Gα and βγ subunit complex of G protein (Gβγ) separates.^7^ Respectively, Gα and Gβγ act on their downstream molecules such as signaling pathway molecules, kinases, or ion channel proteins to perform a range of physiological roles.^4^ Gα has an endogenous GTP-enzyme function that can degrade the associated GTP back into GDP, after which Gα is inactivated and re-forms a complex with Gβγ, and eventually terminates the G protein-dependent signaling.^8^ Therefore, the rate of GTP hydrolysis determines the duration and intensity of G protein activation and physiological function. However, it has been reported that endogenous GTPase activity dependent solely on Gα does not hydrolyze the connected GTP into GDP in time.^9^ Therefore, many other molecules may enhance the function of endogenous GTPase as GTPase activating protein (GAP).^10^

**Figure 1:**
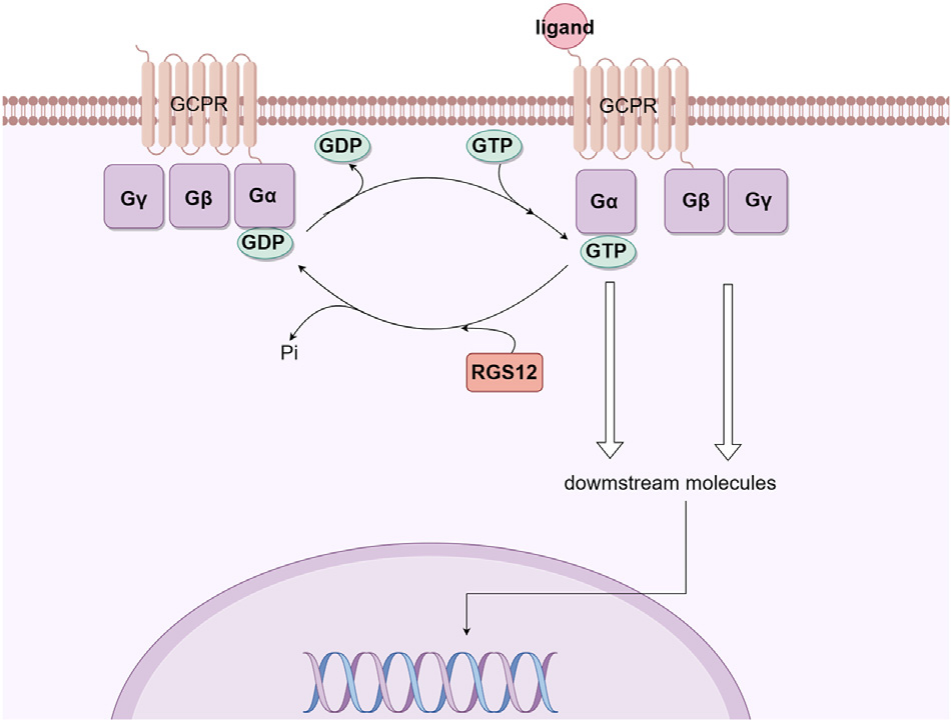
Regulation of GPCR signaling by RGS12. The Gβγ heterodimer serves to couple Gα to the receptor and to inhibit its spontaneous release of GDP. Ligand-occupied, GPCRs stimulate signal onset for Gα subunits, facilitating GDP release, subsequent binding of GTP, and release of the Gβγ dimer. Both the GTP-bound Gα and liberated Gβγ moieties are then able to modulate the activity of downstream molecules. RGS proteins stimulate signal termination by acting as GAPs for Gα, dramatically enhancing their intrinsic rate of GTP hydrolysis. GPCR, G-protein-coupled receptor; RGS, regulator of G-protein signaling; RGS12, regulator of G protein signaling 12; Gβγ, βγ subunit complex of G protein; Gα, alpha subunit of G protein; GDP, guanosine diphosphate; GTP, guanosine triphosphate; GAPs, GTPase-accelerating proteins. This figure was drawn using Figdraw.

Regulator of G protein signaling (RGS) proteins are the largest GAP subfamily discovered so far. They are named for a common RGS domain containing about 120 amino acids, with more than 30 members having been discovered.^11^^,^^12^ The RGS domain enhances the endogenous GTPase activity of Gα, promoting the hydrolysis of GTP to GDP, and exerts inhibitory regulation on the activation of G protein and its downstream signaling pathway^13^^,^^14^ (Fig. 1). It has the characteristic of classical desensitizing receptor signal, thus preventing the overactivation of the downstream cascade reaction of GPCR and maintaining intracellular stability. Furthermore, RGS also regulates the Ca^2+^ signaling pathway, acts as a scaffold protein to bind GPCR receptors with related signaling proteins, and controls the cell membrane's K^+^ and Ca^2+^ channels.^15^

As the largest family member of RGS proteins, RGS12 is widely expressed in the body, which underscores its significance in fine-tuning the specificity and responsiveness of signaling pathways in the context of each tissue microenvironment. Its precise regulation is essential for homeostasis maintenance, adaptive responses to environmental challenges, and preventing the onset of pathological states. Through studying the patterns of RGS12 expression across different tissues, we can gain valuable insights into its role in physiological and pathological processes, and ultimately develop novel therapeutic strategies. Here, we comprehensively review the role of RGS12 in human diseases, such as rheumatoid arthritis, osteoporosis, periodontitis, as well as cancers and nervous disorders. Moreover, we discuss the possibility of RGS12 as a precise therapeutic target for these diseases.

### RGS protein family and RGS12

The α subunit of G protein contains four subfamilies: Gαs, Gαi/o, Gαq/11, and Gα12/13.^16^, ^17^, ^18^, ^19^ So far, most of the identified RGS family members have specific regulatory effects on Gαi/o and Gαq/11, and RhoGEF is the only RGS family member with GAP activity on Gα12/13.^15^ Different RGS molecules have a variety of specific molecular structures on both sides of the core RGS domain. These functional domains of family members affect the specificity of substrate regulation and determine the intracellular localization of molecules and other functional actions. Based on their molecular structures and functions, the family is divided into seven subfamilies: A/RZ, B/R4, C/R7, D/R12, E/RA, F/GEF, and G/GRK^20^^,^^21^ (Table 1). Among them, A/RZ, B/R4, C/R7, and D/12 are the classical RGS subfamilies. The A/RZ subfamily consists of three members, RGS17, RGS19, and RGS20, all of which are small proteins with a cysteine string domain located near the N-terminal, which is used mainly to regulate the membrane localization. The B/R4 subfamily includes RGS1, RGS2, RGS3, RGS4, RGS5, RGS8, RGS13, RGS16, RGS18, and RGS21. Most members of the B/R4 subfamily belong to small RGS molecules with 20–30 KD in size.^19^^,^^22^ RGS3 has a PDZ (PSD-95/Discs large/ZO-1 homology) structural domain at the N-terminal, which is different from other members of the B/R4 subfamily. In addition to the specific action of RGS2 on Gαq, the GTPase activating proteins of other members in the B/R4 subfamily are not selective to Gαi/o or Gαq.^23^^,^^24^ The C/R7 subfamily consists of RGS6, RGS7, RGS9, and RGS11, which are mainly expressed in the nervous system, regulating a variety of nervous system functions including vision, memory, motor control, reward behavior, and harm perception.^25^ The GTPase activating proteins of the C/R7 subfamily mainly target Gαi/o. The members share both a DEP (Disheveled, EGL-10, Pleckstrin) domain and a GGL (G gamma-like) domain at the N-terminal in addition to the RGS domain. D/R12 subfamily members are relatively complex, including RGS10, RGS12, and RGS14. Due to the lack of specific domains to regulate GAP, RGS10 exerts inhibitory regulatory effects on Gαi/0 and Gαq.^26^ RGS12 and RGS14 only act as GAP for Gαi/o. In addition, RGS12 and RGS14 possess a unique GoLoco (Gαi/o-Loco) interaction motif at the C-terminal and a tandem RAS-binding domain (RBD), which inhibits both the dissociation of GDP from Gαi and GTP binding with Gαi.

**Table 1:** RGS subfamily and related structural domains.

Subfamilies		Members	Relevant domains
Classical RGS subfamily	A/RZ	RGS17,19,20	RH, CYS
	B/R4	RGS1, 2, 4, 5, 8, 13, 16, 18, 21, RGS3	RH, A-helix, RH, PDZ,
	C/R7	RGS6, 7, 9, 11	RH, DEP, GGL
	D/R12	RGS10, 12,14	RH, PDZ, PTB, RBD, GoLoco
Nonclassical RGS subfamily	E/RA	Axin Axin2	RH, β-cat, GSK3β, DAX, RH, DAX
	F/GEF	P115-RhoGEF, GRK2, RGS22	RH, DH, PH
	G/GRK	GRK1,4,5,6,7	RH, Kinase, PH

RGS12, the largest family member, has 1376 amino acids. In addition to the central RGS domain, RGS12 also contains the PDZ domain, the phosphotyrosine binding (PTB) domain, RBD, and the GoLoco motif.^27^ On the one hand, the RGS domain has GAP activity, accelerating the hydrolysis of GTP. On the other hand, the GoLoco sequence slows down the nucleotide exchange of G protein, plays the role of guanine nucleotide dissociation inhibitor (GDI), and significantly reduces the signal transmission rate.^28^ The N-terminal of RGS12 contains a PDZ domain, and the C-terminal contains a PDZ binding module.^29^^,^^30^ The PDZ domain consists of about 90 amino acids, containing a conserved sequence of alanine-isoleucine-alanine-proline, which can be used as a scaffold to bind to proteins containing the PDZ binding module. When there is no signal stimulation, the PDZ domain of the N-terminal binds to its PDZ binding module of the C-terminal to inhibit the GAP activity. The interleukin-8 receptor is a Gαi-coupled GPCR belonging to the CXCR2 (C-X-C motif chemokine receptor 2) family.^15^ The PDZ domain of RGS12 can act specifically on the interleukin-8 receptor. When CXCR2 binds to the PDZ domain, the RGS domain is exposed, exerting its GAP activity of binding to the Gαi/o subunit. RGS12 can also terminate Ca^2+^ channel signaling mediated by gamma-aminobutyric acid type B through its PTB domain binding to phosphotyrosine on the subunit 1B of Ca^2+^ channel.

## RGS12 in bone tissue

### RGS12 in bone homeostasis

Bone homeostasis is strictly regulated by the balance between osteoblasts (OBs) and osteoclasts (OCs).^31^^,^^32^ Both OBs and OCs are derived from mononuclear/macrophage hematopoietic lineages. The increasing activity of OCs would lead to loss of bone mass and result in fractures in patients.^33^ OBs are the only cell components for bone formation and repair.^34^ Therefore, regulating the activity and differentiation of OBs and OCs is critical for maintaining bone homeostasis.

The expression of RGS12 increases gradually during OC differentiation, which is crucial for OC differentiation. Yuan et al found that RGS12 affected the terminal differentiation of OCs by regulating the receptor activator of NF-κB ligand (RANKL)/Ca^2+^/activated T nuclear factor (NFAT) 2 signaling pathway.^35^ Silencing of RGS12 in hematopoietic cells or OC precursors blocks OC differentiation and function, leading to increasing bone mass, which could be used to prevent postmenopausal osteoporosis and inflammation-induced bone loss.^35^ Yang et al also found that phosphorylation of phospholipase Cγ (PLCγ) was a key component of the RANKL-induced Ca^2+^ oscillation-NFAT2 pathway.^36^ After being activated by the activator, RGS activates PIP3 (phosphatidylinositol (3,4,5)-trisphosphate) through its RGS domain to exert the GAP effect, while Ca^2+^-CaM can promote the activation of PLC, IP3, and Ca^2+^ release to relieve the GAP effect. Silencing of RGS12 disrupts PLCγ phosphorylation, blocks Ca^2+^ oscillations, and inhibits NFAT2 expression and OC differentiation, suggesting that RGS12 induces PLCγ activation and Ca^2+^ oscillations through GAP activity in its RGS domain.^36^

**Figure 2:**
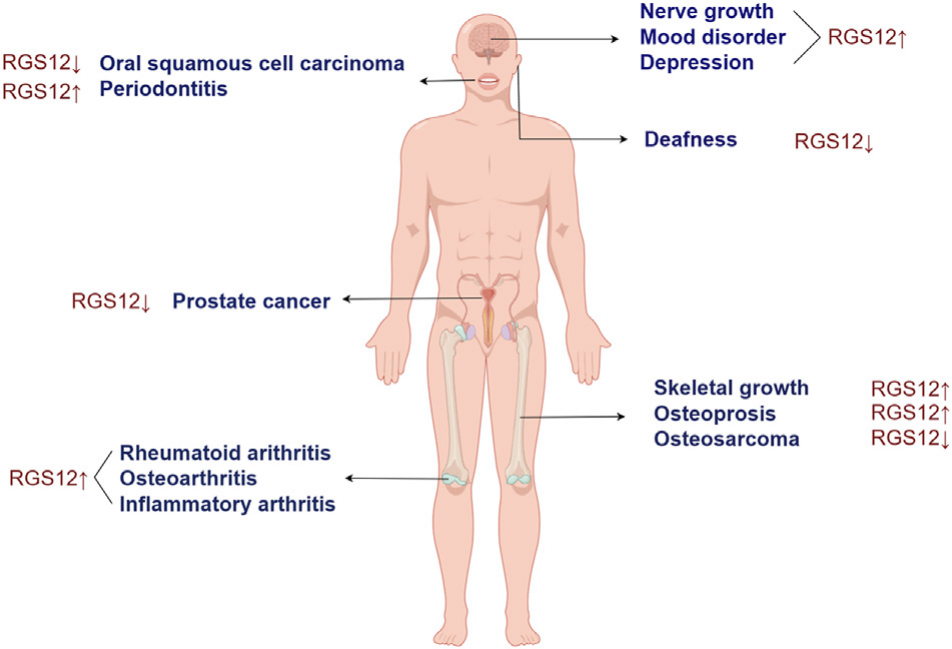
Overview of RGS12 expression in various human diseases. Arrows indicate the regulation of the respective diseases. The main concern is the regulation of RGS in the diseases described in this review (brain, oral cavity, ear, prostate gland, bone, and joint). RGS, regulator of G-protein signaling; RGS12, regulator of G protein signaling 12. This figure was drawn using Figdraw.

**Figure 3:**
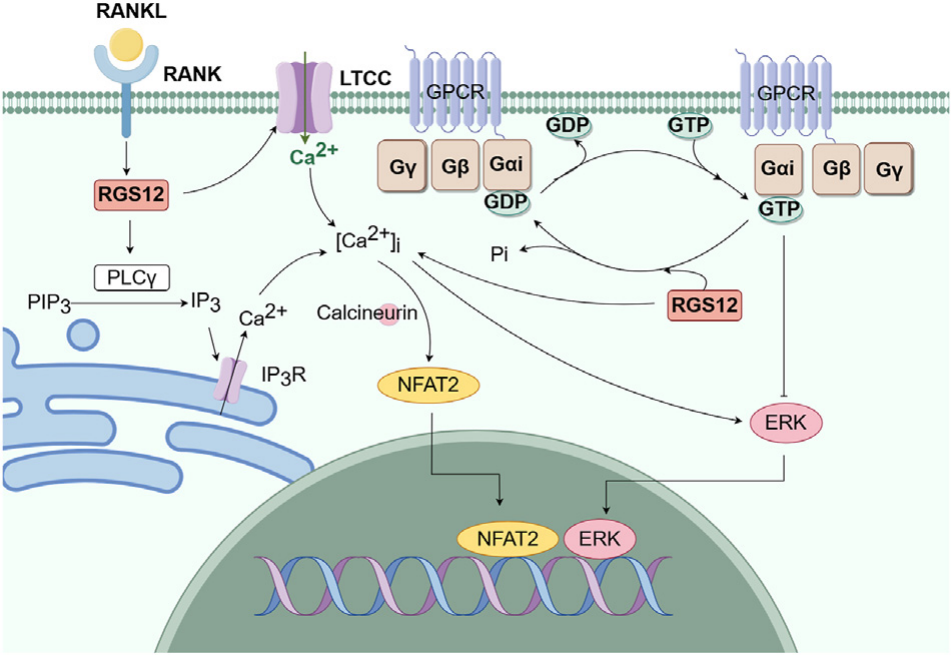
The relevant signaling pathways of RGS12 in bone homeostasis. In OCs, RGS12 regulates the RANKL/Ca^2+^/NFAT2 signaling pathway to promote the terminal differentiation of OCs. Meanwhile, in OBs, RGS12 promotes the activity of GTPase against the Gαi subunit, enhancing the inhibition of Gαi mediated signaling and resulting in osteolytic phenotype. RGS12, regulator of G protein signaling 12; OCs, osteoclasts; RANKL, receptor activator of NF-κB ligand; NFAT2, activated T nuclear factor 2; OBs, osteoblasts. This figure was drawn using Figdraw.

OB differentiation and proliferation are also affected by Ca^2+^ signaling control gene expression. Li et al found that Gαi-ERK signaling was one of the pathways through which RGS12 played a regulatory role in osteogenesis.^37^ Gαi-mediated overactivation of signals acts as a negative regulator of OB differentiation by inhibiting adenylyl cyclase (AC) and ultimately leads to reduced trabecular bone formation, whereas the Gαs-AC-cAMP signaling pathway promotes OB differentiation. Loss of RGS12 reduces the activity of GTPase against the Gαi subunit, lessening the inhibition of Gαi mediated signaling and resulting in osteopenic phenotype^37^ (Figure 2, Figure 3).

### RGS12 in skeletal growth

The growth of bone has two forms, intrachondral osteogenesis and intramembrane osteogenesis.^38^ The four limbs' bone and trunk bone are mainly endochondral osteogenesis. Growth plates, also known as epiphyseal plates, are cartilaginal tissues located between the epiphyseal and the backbone of the bone that lengthen the long bone through chondrogenesis and intrachondral ossification.^39^ Composed mainly of chondrocytes and extracellular matrix, the growth plate is a highly metabolic tissue that produces energy for bone growth. According to the differentiation stage of the chondrocytes, the growth plate can be divided into four layers: the resting zone, the proliferating zone, the prohypertrophic zone, and the hypertrophic zone.^40^ In the hypertrophic region, spherical chondrocytes engulf the extracellular matrix and complete final differentiation. Some chondrocytes differentiate into OBs, while some apoptotic cells form the skeleton, and then blood vessels, OBs, and OCs enter, and finally, ossification becomes bone.^41^ Mitochondria are the primary sites of energy generation, and their homeostasis is crucial for bone growth.^42^ The energy metabolism of chondrocytes in the hypertrophic region was very active, and the content of mitochondrial protein increased, as well as the volume of mitochondria.^43^

Mitochondria provide energy to cells through oxidative phosphorylation, and complex V, a key enzyme in the respiratory chain steps, contains the F0 and F1 domains. The F1 domain uses an electrochemical gradient to convert ADP to ATP.^44^ ATP5A1 is an important component of complex V, which functions as ATP synthase and can affect the regulation of energy production, reactive oxygen species (ROS) production, and programmed cell death.^45^ Yuan et al found that RGS12 could bind to tyrosine kinases such as TykA, thus promoting Tyr phosphorylation of ATP5A in mitochondria, producing ATP, and promoting endochondral osteogenesis. Thus, RGS12 is involved in maintaining endochondral osteogenesis by regulating ATP5A.^27^

### RGS12 in osteoporosis

Osteoporosis is a common senile disease in clinical practice.^46^ It is characterized by decreased bone mass and degeneration of bone microstructure, leading to an increased risk of fracture. Bone remodeling is accomplished by the synergistic action of OBs and OCs. Bone deficiency disorders such as osteoporosis are typically characterized by increased OC activity, which leads to net bone loss.^47^

ROS are a normal by-product of cell metabolism.^48^ According to the aging theory, the damage caused by ROS gradually accumulates and eventually manifests as age-related degenerative diseases. Callaway et al found that RANKL-induced ROS was an integral part of OC differentiation.^48^ ROS is a double-edged sword. High ROS concentrations can damage OC, while low ROS concentrations are involved in signal transduction in OC differentiation, including RANKL-dependent activation of mitogen-activated protein kinases (MAPKs), PLCγ, nuclear factor κB (NF-κB), and Ca^2+^ oscillations.^49^ All of these contribute to the activation of NFAT, a major regulator of OC differentiation.^50^ Blocked ROS production inhibited OC differentiation.^51^ Andrew Ying Hui Ng et al found that RGS12 promoted the production of ROS by inhibiting Nrf2 and its target antioxidant genes, thus promoting the formation of OCs. Therefore, overexpression of RGS12 increases the number and size of OCs and improves bone resorption activity.^52^ Therefore, RGS12 is an important regulator of OC differentiation and function.

Thus, the results to date suggest RGS12 a role in regulating bone remodeling and bone loss through the interaction with the inflammation-related pathway, such as NF-κB, as well as the energy metabolism pathway, like TykA/ATP5A. By targeting RGS12 specifically, it is possible to modulate osteoporosis and inflammation-induced bone loss at a fundamental level.

## RGS12 in arthritis

### RGS12 in rheumatoid arthritis

Rheumatoid arthritis is a chronic systemic inflammatory disease in which irreversible bone damage eventually leads to joint dysfunction and even deformity.^53^ Each cell is involved in the process of bone destruction and interacts with each other to form a complex network. Among them, macrophages can produce a large number of inflammatory factors, such as lipopolysaccharide, tumor necrosis factor-alpha (TNF-α), and prostaglandin E2 (PGE2), which destroy the cartilage and bone around the joint, resulting in joint pain and swelling in patients.^54^^,^^55^

Yuan et al found that RGS12 expression increased significantly in rheumatoid arthritis patients by comparing the GEO database of rheumatoid arthritis patients and healthy people.^56^ RGS12 promotes NF-κB activation through its PTB domain binding to NF-κB. At the same time, PGE2 increases RGS12 expression, NF-κB activation, and nuclear translocation via prostaglandin E2 receptor EP4. Activated NF-κB can further promote the production of PEG2, as well as the expression of RGS12. Therefore, the PTB domain of RGS12 is a potential therapeutic target for rheumatoid arthritis, while PGE2/EP4 signaling and RGS12/NF-κB signaling form positive feedback for inflammation regulation.^56^

### RGS12 in osteoarthritis

Osteoarthritis is a common degenerative joint disease.^57^ The main pathological manifestations of osteoarthritis are chondrofibrosis, softening, ulcer formation and absence, subchondral osteosclerosis, osteophyte formation, synovitis, and synovitis.^58^ The main clinical symptoms are progressive joint pain and dysfunction.^59^ The increase of inflammatory cells and inflammatory factors in osteoarthritis leads to synovitis and cartilage destruction. Among them, macrophages can secrete pro-inflammatory cytokines, such as IL-1β, IL-6, and TNF-α, and participate in the progression of osteoarthritis.^60^

**Figure 4:**
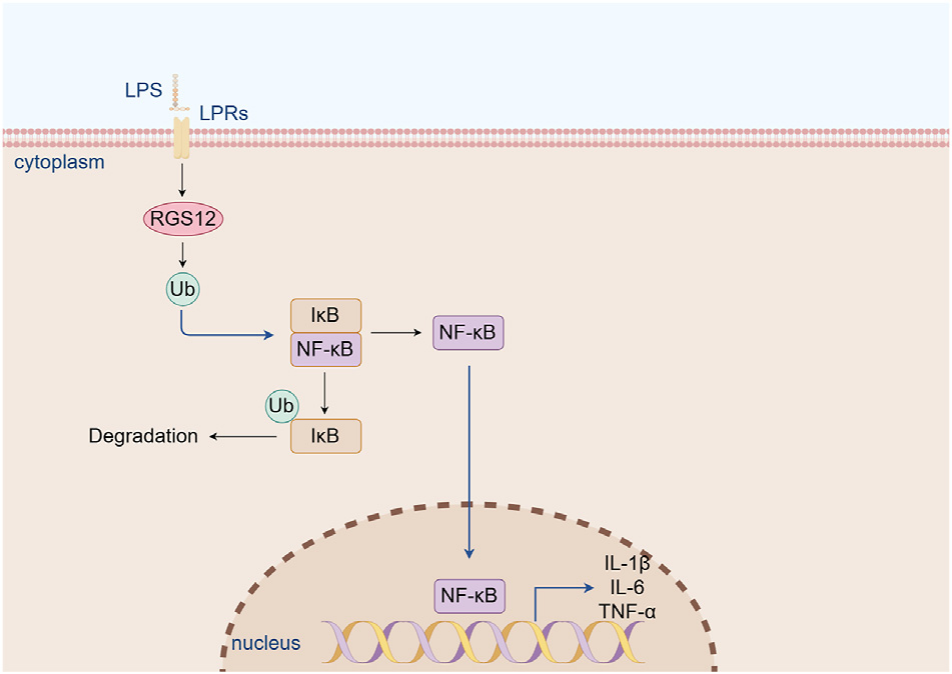
The relevant signaling pathways of RGS12 in arthritis. RGS12 promotes the degradation of IκB by enhancing the ubiquitination. Due to the degradation of IκB, NF-κB translocates into the nucleus and further promotes the gene expression of cytokines such as IL-1β, IL-6, and TNF-α during inflammation. RGS12, regulator of G protein signaling 12; IκB, inhibitory kappa B; NF-κB, nuclear factor κB; IL, interleukin; TNF-α, tumor necrosis factor-alpha. This figure was drawn using Figdraw.

NF-κB is a hub signaling protein downstream of toll-like receptor-4 (TLR4) protein responsible for mediating inflammation.^61^ In the absence of pathogenic agent invasion, the dimer of NF-κB can be combined with I-κB (inhibitory protein) to form a trimer to inactivate the protein. The ubiquitin-proteasome pathway plays a key role in the NF-κB pathway.^62^ Upon stimulation, I-κB is phosphorylated and subsequently ubiquitinated and degraded by the 26S proteasome, thereby allowing NF-κB to be transported to the nucleus, promoting the expression of inflammatory mediator genes, playing an inflammatory response biochemical signaling pathway, leading to impaired metabolic activity of chondrocytes, which in turn promotes the progression of joint pain and dysfunction.^63^^,^^64^ Yuan et al found that RGS12 could affect lipopolysaccharide-induced ubiquitination and I-κB degradation. Increased expression of RGS12 in macrophages promotes ubiquitination and NF-κB activation. Knockout of RGS12 in macrophages can reduce the expression of IL-1β, IL-6, and TNF-α, and reduce cartilage destruction in osteoarthritis^65^ (Fig. 4).

### RGS12 in inflammatory arthritis

Synovial fibroblasts are essential for arthritis progression and ciliogenesis in synovial fibroblasts is positively related to the development of arthritis.^66^ Yuan et al found that RGS12 maintained ciliogenesis in synovial fibroblasts.^67^ RGS12 interacts with MYC-binding protein 2 to enhance its ubiquitination activity, resulting in the degradation of kinase family member 2A (KIF2A) protein in synovial fibroblasts, which increases the length and number of cilia. The increased ciliogenesis results in the activation of synovial fibroblasts, which leads to joint swelling and cartilage damage. Loss of RGS12 results in a significant reduction in cilia generation, adhesion, migration, and synovial fibroblast secretion.^67^ Therefore, RGS12 may be a potential drug target for treating inflammatory arthritis.

Thus, the results to date suggest RGS12 plays a critical role in the pathogenesis of arthritis, including inflammatory arthritis, osteoarthritis, and rheumatoid arthritis, through mediating the dysfunction of macrophages, fibroblasts, and endothelial cells. These results also provide evidence for the relevance of RGS12 in promoting inflammation and regulating immune-related signaling pathways. It is possible to modulate arthritis at a fundamental level by targeting RGS12 specifically. However, it is important to note that the therapeutic potential of targeting RGS12 is still in its infancy, and future research is needed to fully understand the consequences of modulating its activity *in vivo*.

## RGS12 in cancer

### RGS12 in oral squamous cell carcinoma

Oral squamous cell carcinoma (OSCC) is one of the most common malignant tumors of the head and neck.^68^^,^^69^ Patients with OSCC treated with traditional methods tend to have a poor prognosis, are prone to primary recurrence and local lymphatic metastasis, and have a low 5-year survival rate.^70^^,^^71^ Therefore, it is particularly important to find a new target for the diagnosis and treatment of OSCC and to achieve a good prognosis.

Recently, Fu et al conducted an immunohistochemistry analysis of human OSCC tissues and found that the expression of RGS12 in OSCC tissues was significantly reduced. Further analysis showed that the expression of RGS12 was correlated with the TNM stage and pathological grade of OSCC.^72^ Phosphatase and tension homologs (PTEN) are important inhibitors of human cancer^73^ with C-terminal PDZ domain-binding MOBS (PBM) that can be recognized by scaffolding proteins and regulatory proteins and bind specifically to the PDZ domain to suppress tumor growth.^74^, ^75^, ^76^ RGS12 interacts with PTEN through the PDZ domain, up-regulating phosphorylation and sumoylation of PTEN, thereby inactivating the protein kinase B (AKT)/mammalian target of rapamycin (mTOR) signaling pathway and suppressing OSCC growth.^72^ Thus, RGS12 is an essential tumor suppressor and might be a potential therapeutic target for OSCC.

OSCC, as a type of cancer, is located in a complex tumor microenvironment.^77^ Tumor-associated macrophages are important components of the tumor microenvironment and the main tumor-infiltrating white blood cells in most cancers, associated with cancer immunosuppression and immunotherapy.^78^ In different environments, tumor-associated macrophages could differentiate into M1-like and M2-like subtypes.^79^ In patients with oral cancer, M1 macrophages, not M2 macrophages, are conducive to prognosis survival.^80^ Therefore, the phenotype and functional changes of macrophages are crucial for the choice of treatment and the assessment of prognosis in patients with OSCC.

Previously, Singh et al found that primary cilia are important organelles of bone marrow monocytes, whose assembly and length of cilia change in different environments, thus regulating the phenotype and function of macrophages.^81^ Primary cilia are critical for tumor progression and microenvironment. RGS12 promotes M1 macrophage polarization. Yuan et al found that RGS12 could regulate ciliogenesis by analyzing the GenomeRNAi database and might be a key regulator for tumor-associated macrophage polarization to M1 phenotype.^82^ RGS12 enhances the phosphorylation of Myc-binding protein 2 (MYCBP2) to degrade the ciliary protein KIF2A and cilia disassembly, which promotes tumor-associated macrophage polarization into M2 macrophages. Thus, the RGS12/MYCBP2 pathway is a potential therapeutic target for OSCC.

### RGS12 in osteosarcoma

Osteosarcoma is a malignant tumor originating from bone tissue, characterized by tissue heterogeneity, local invasion, and rapid metastasis.^83^ It is most common in children and adolescents and has a high rate of disability.^84^

The Hippo pathway is a highly evolutionarily conserved protein kinase signaling pathway, which regulates cell proliferation, apoptosis, and organ morphology and size through the regulation of downstream transcriptional co-activator Yes-associated protein (YAP).^85^, ^86^, ^87^ As transcriptional coactivators, YAPs do not have a DNA-binding domain, and they promote downstream by interacting with the transcription factor TEADs, significantly enhancing Ezrin expression, thereby leading to the growth and progression of osteosarcoma and lung metastasis.^88^ Li et al found that RGS12 binds to YAP through its PDZ domain and inhibits the nuclear translocation of YAP and the expression of Ezrin in osteosarcoma models, consequently inhibiting osteosarcoma growth.^89^ At the same time, RhoA participates in the Hippo pathway, and GPCR transmits extracellular signals by coupling heterotrimer Gα12/13 protein to activate RhoA, leading to YAP activation.^90^ Thus, RGS12 might suppress osteosarcoma growth by regulating the RhoA/YAP/TEAD1/Ezrin signaling pathway.^89^

### RGS12 in prostate cancer

Prostate cancer (PCa) is the most common cancer among men worldwide and the fifth leading cause of death among men with malignant tumors.^91^ African American men have significantly higher rates of PCa than European American men and are twice as likely to die from PCa.^92^

**Figure 5:**
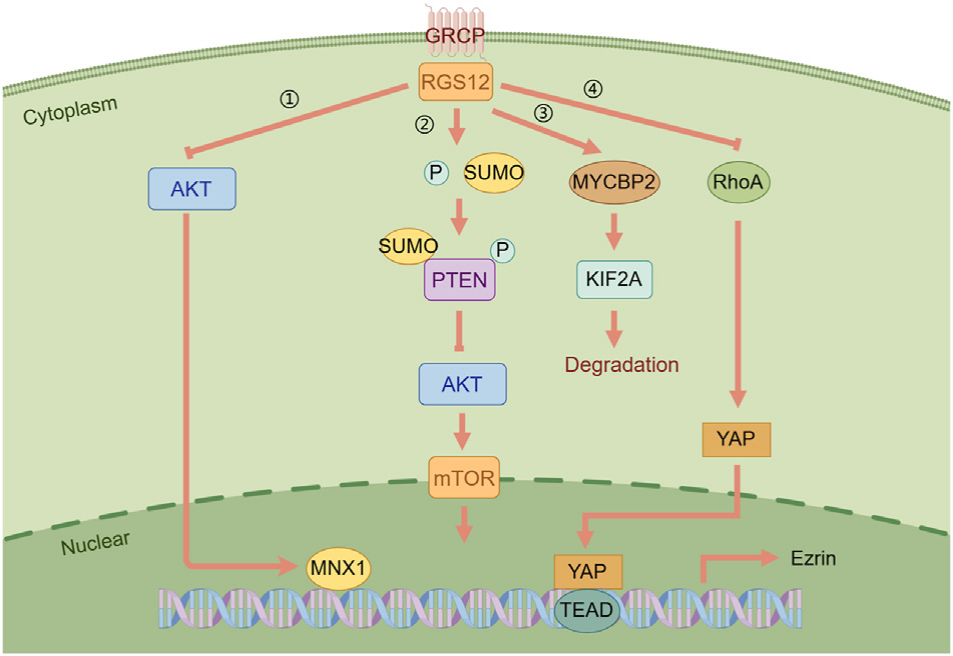
The signaling pathways of RGS12 in tumor. (i) RGS12 negatively regulates the level and activity of AKT protein to inhibit the expression of MNX1. (ii) RGS12 interacts with PTEN through the PDZ domain, up-regulating phosphorylation and sumoylation of PTEN, thereby inactivating the AKT/mTOR signaling pathway and suppressing OSCC growth. (iii) RGS12 enhances the phosphorylation of MYCBP2 to degrade the ciliary protein KIF2A. (iv) RGS12 binds to YAP and inhibits the nuclear translocation of YAP and the expression of Ezrin. RGS12, regulator of G protein signaling 12; AKT, protein kinase B; MNX1, motor neuron and pancreas homeobox 1; PTEN, phosphatase and tension homolog; PDZ, PSD-95/Discs large/ZO-1 homology; mTOR, mammalian target of rapamycin; OSCC, oral squamous cell carcinoma; MYCBP2, Myc-binding protein 2; KIF2A, kinase family member 2A; YAP, Yes-associated protein. This figure was drawn using Figdraw.

Wang et al found that the expression of RGS12 in PCa cells was lower than that in benign prostate epithelial cells.^93^ At the same time, RGS12 was preferentially deleted in African American PCa compared with European American PCa, and RGS12 transcription levels were relatively low. As a carcinogenic transcription factor, MNX1 (motor neuron and pancreas homeobox 1) enhances lipid synthesis and is associated with cancer aggressiveness, which is significantly increased in African American PCa. Gene expression data showed that overexpression of RGS12 could strongly inhibit the expression of MNX1 in African American PCa tissues. Meanwhile, AKT can regulate the expression of MNX1, while RGS12 negatively regulates the expression level and activity of AKT protein.^93^ Thus, RGS12 might be a tumor suppressor gene in PCa (Fig. 5).

Thus, the above-mentioned studies suggest that RGS12 plays a role in tumors by regulating the proliferation, invasion, and metastasis of cancer cells, as well as the immune microenvironment. Given the differential expression of RGS12 in various tumors and the different effects in different tumors, it can be speculated that RGS12 may also play a role in some other cancers, such as lung cancer. Targeting RGS12 may provide a new strategy for anti-tumor therapy. Furthermore, developing potent inhibitors or activators of RGS12 will be crucial for translating these observations into real-world clinical practice.

## RGS12 in the nervous system

### RGS12 in nerve growth

GPCRs are one of the most abundant protein families in the nervous system.^94^ Signaling pathways mediated by G proteins in the nervous system are critical for brain development and normal nerve conduction.^95^^,^^96^

Loco is expressed in lateral glial cells in the developing embryonic central nervous system and is required for correct glial cell differentiation. RGS contains a GoLoco domain which could interact with Loco in surface glia.^27^ Melinda et al found that RGS12 could regulate glial cell growth and differentiation, through binding to nerve growth factor receptor TrkA, activating H-Ras, B-Raf, and MEK2, and promoting ERK transferring to the nucleus.^97^ Down-regulated expression of RGS12 inhibits nerve growth factor-induced axonal growth in primary dorsal root ganglion neurons.^97^ Thus, RGS12 plays a key receptor-selective role in coordinating RAS-dependent signals that are necessary to promote and maintain neuronal differentiation.

### RGS12 in mood regulation

The dorsal raphe nucleus is located on the ventral side of the midbrain aqueduct and is fan-shaped with glutamate, dopamine, gamma-aminobutyric acid, and 5-hydroxytryptamine (5-HT).^98^ 5-HT neurons, which account for about 2/3, are mainly located in the abdominal and ventrolateral regions and are involved in regulating a variety of functional states, such as pain, anxiety, reward, and sleep-wake.^99^ Thus, 5-HT-ergic neuronal dysfunction is associated with neuropsychiatric disorders.^100^ Amphetamine drugs, also known as amphetamines, are a class of neurostimulants. Representative drugs including methamphetamine, 3, 4-methylenedioxymethamphetamine (MDMA), binding DA transporter (DAT), and 5-HT transporter (SERT) improve the concentration of 5-HT in the body.^101^ Recent studies have found that MDMA can be used as an adjunct to psychotherapy, especially for post-traumatic stress disorder.^102^

RGS12 is highly expressed in the embryonic nervous system and shows significant protein expression in the serotonin-5-HT projective regions of the adult mouse brain such as the cortex, hippocampus, striatum, and midbrain.^103^^,^^104^ Allison et al found that in RGS12 deficient mice, acute administration of DAT-targeted psychostimulants (AMPH, cocaine) was associated with reduced stimulation of excessive exercise.^105^ In addition, these mice showed brain region-specific changes in DAT expression and function. Loss of RGS12 is associated with reduced excessive movement at low doses of MDMA, increased brain region-specific SERT expression and 5-HT uptake, and increased anxiety-like and antidepressant-like behaviors. Thus, RGS12 expression is necessary to maintain the normal function of specific 5-HT energy regions and circuits in adult mice.^105^

Opioid receptors are widely found in the human body and are highly expressed in regions of the central nervous system associated with mood and pain, regulating neural circuits for reward-seeking, motivation processing, stress response, and pain sensitivity.^106^ Endogenous opioid receptors belong to the GPCR superfamily of rhodopsin.^107^^,^^108^ Kappa opioid receptor (KOR) agonists can reduce pain and produce drug reward-behavioral outcomes but can cause adverse reactions such as aversion, anxiety, and irritability.^109^^,^^110^ The clinical effects of KOR agonists are mediated by G protein signaling, while adverse reactions are caused by β-statin signaling.^111^ Joshua et al found that RGS12 can weaken the G protein signaling pathway and enhance β-block protein signaling, which is manifested by enhanced pain and aversion. In contrast, RGS12-deficient mice showed decreased emotions such as aversion induced by KOR agonists.^100^

### RGS12 in depression

Depression is a mental disorder characterized by persistent depression, loss of interest, sleep disorder, loss of appetite, and low libido.^112^ The results show that the pathophysiological process of depression is related to neuronal damage, neurotransmitter changes, and abnormal synaptic plasticity caused by inflammation and oxidative stress.^113^ The brain has a high metabolic rate and antioxidant capacity and is particularly vulnerable to oxidative damage.^114^ Therefore, oxidative stress may be associated with depression.

Studies have found that certain miRNAs are associated with the development of central nervous system diseases. There are also some changes in miRNA levels in the brains of depressed people.^115^ Tian et al used high-throughput sequencing and the Kyoto Encyclopedia of Genes and Genomes (KEEG) database to screen out differentially expressed miRNAs in depressed rat models and normal rats.^116^ They found that miR-204-5p was significantly reduced in the hippocampal dentate gyrus region in the depressed rat model and is involved in regulating oxidative stress levels in the brain. RGS12 is one of its downstream target genes. Down-regulation of miR-204-5p leads to increased expression of RGS12, oxidative damage, and inflammation in the hippocampal dentate gyrus region through activation of Nrf2/NF-κB signaling pathway, and increases depressive symptoms.^116^

As depicted above, RGS12, highly expressed in the central nervous system, is implicated in the modulation of GPCR signaling, which is crucial for neuronal communication. It may play a role in neuroprotection, as suggested by studies showing its up-regulation to promote glial cell growth and differentiation in response to neuroprotective stimuli. It is also involved in the regulation of mood regulation and depression. However, the precise functions of RGS12 in the central nervous system are yet to be fully elucidated and further investigation remains to be done.

### RGS12 in periodontitis

Chronic periodontitis is a chronic inflammatory disease occurring in periodontal supporting tissues, mostly caused by periodontal pathogens and immune system imbalance.^117^ It is a common clinical oral disease with an incidence of 35 %–50 %. Chronic periodontitis not only damages alveolar bone and periodontal soft tissue but also leads to tooth loosening and loss.^118^ When it develops into severe periodontitis, it also increases the risk of a variety of systemic diseases and hurts body health.^119^ The disorder of immune response is closely related to the occurrence of chronic periodontitis. Macrophages are inherent immune cells and an important part of innate immunity. The polarization of macrophages under environmental stimulation is closely related to the occurrence and development of chronic periodontitis. In an inflammatory environment, macrophages can differentiate into OCs and dissolve alveolar bone, leading to bone loss.^120^

In an inflammatory environment, RGS12 is activated and transported to the nucleus, further promoting inflammation.^56^ Yuan et al found that RGS12 had the highest expression level in monocytes compared with other immune cells.^121^ RGS12 can promote the polarization of M1 macrophages. RGS12 leads to bone erosion through the polarization of macrophages and the release of inflammatory factors through the GPCR and NF-κB pathways and is a potential therapeutic target for chronic periodontitis.^121^

### RGS12 in the auditory system

Hair cells in the sensory organ of the inner ear are mechanoreceptors.^122^ Hair cells detect sound, body position, and head movement through the stereociliary bundle or hair bundle on the tip.^123^ Inhibition of alpha-class G proteins (GNAI1, GNAI2, and GNAI3; GNAI or Gαi) is essential for hair bundle morphogenesis and sensory function.^124^ GNAI/Gαi binds to scaffold G protein signaling regulator 2 (GPSM2) to form conserved polar complexes to regulate cytoskeletal tissue. GPSM2 keeps GNAI binding GDP. Anil et al found that RGS12 was indispensable for GPSM2-GNAI polarization of the hair cell top membrane and mechanical sensation.^124^ GPSM2 and RGS12 share the GoLoco motif that stabilizes GNAI(GDP), and GPSM2 is superior to RGS12 in binding to GNAI. A third class of regulators specific to Gαi/GNAI are GDIs. GDI binds to GNAI through its GoLoco motif and keeps GNAI in an inactive state combined with GDP.^125^ GPSM2 is a conservative partner of GNAI during directed cell division.^126^ During cell division, the cortical GPSM2-GNAI(GDP) polarity complex directs the mitotic spindle by recruiting effector agents that exert force on the stellate microtubules. N-terminal acylation of GNAI is essential for anchoring GPSM2 and polarizing effectors on cell membranes. The GoLoco motif carried by RGS12 has GDI activity.^127^ Thus, RGS12 can not only bind to GNAI through its namesake RGS domain, thereby acquiring GAP activity but also regulate GDI activity through its GoLoco motif. Thus, both RGS12 and GPSM2 can combine and isolate GNAI(GDP). RGS12 is essential for the generation and polarization of GPSM2-GNAI complexes on adjacent apical membranes; that is to say, RGS12, a novel deafness protein, is an indispensable link of GPSM2-GNAI and heterotrimer G protein signaling in hair cells.

### Potential mechanisms for the regulation of RGS12 expression

As mentioned above, many studies have reported the molecular function of RGS12 in multiple tissues, while the regulatory mechanism of tissue-specific expression of RGS12 is rarely reported. Recently, some studies demonstrated the molecular signaling pathway involved in the expression of RGS12. For instance, a rodent study of inflammatory pain demonstrated that overexpression of COX2 and PGE2 in macrophages enhanced the expression of RGS12, in which PGE2 regulated RGS12 expression through the G-protein-coupled receptors EP2 and EP4.^128^ The authors also found that RGS12 expression was regulated by NF-κB (p65) in a transcriptional manner.^56^ Moreover, RGS12 was proved as a downstream target of microRNA-204-5p in a stress-induced pathological model,^4^ and circular RNA hsa_circ_0006091 in hepatocellular carcinoma.^129^

DNA methylation in CpG islands or at the first exon can lead to gene silencing. Nhan et al reported that epirubicin altered DNA methylation profiles related to cardiotoxicity, and found that RGS12 was hypo-methylated and up-regulated on the transcriptome level in a part of the samples exposed to epirubicin due to different times of exposure.^130^ Recently, DNA methylation of RGS12 was demonstrated to be significantly associated with the prognostic outcomes of gastric cancer.^131^ Moreover, the DNA methylation level of RGS12 was observed to decrease with its mRNA level up-regulated in the restricted-diet-fed pig endometrium and embryos.^132^

According to the central dogma of molecular biology, the tissue-specific expression of RGS12 may be governed by a complex interplay of transcriptional, epigenetic, post-transcriptional, and post-translational mechanisms. Dysregulation of these mechanisms can lead to the alteration of RGS12 expression and function. Therefore, further investigation into the molecular mechanisms underlying RGS12's tissue-specific expression and its role in disease pathogenesis holds great promise for developing novel therapeutic strategies.

## Conclusions and prospects

In conclusion, many life activities are finely regulated by RGS12, which is widely expressed in human cells and tissues. RGS12 has been shown to play a role in the pathogenesis of many human diseases, including osteoporosis, fracture repair, arthritis, tumors and cancers (*e.g.*, osteosarcoma, PCa, OSCC), neurological disorders (*e.g.*, anxiety, depression), periodontitis, and hearing disorders. The upstream and downstream regulatory mechanisms of RGS12 have been reported in many ways. RGS12 is a key node in pathogenesis and a biomarker for the clinical features of many diseases, making it a possible target for the precise treatment of these diseases.

The pathogenesis of many other diseases may also be related to RGS12, which requires more extensive research in the future. It is found that RGS12 is highly expressed in fibroblasts, one of the important cells that produce extracellular matrix, and plays an important role in tissue fibrosis or wound scarring, which needs further study. At present, attempts have been made to develop drugs targeting RGS protein,^133^ which have shown good prospects both *in vitro* and *in vivo*, and drug development based on RGS12 will be very meaningful and urgent.

## CRediT authorship contribution statement

**Min Jiang:** Investigation, Writing – original draft, Writing – review & editing. **Hongmei Li:** Writing – original draft. **Qiong Zhang:** Data curation, Investigation. **Tongtong Xu:** Data curation, Investigation. **Le Huang:** Investigation, Resources. **Jinghong Zhang:** Investigation, Resources. **Huiqing Yu:** Conceptualization, Supervision, Writing – review & editing. **Junhui Zhang:** Conceptualization, Funding acquisition, Supervision, Writing – review & editing.

## Funding

This work was supported by the National Natural Science Foundation of China (No. 82100889), China Postdoctoral Science Foundation (No. 2024M753868), Chongqing Doctor “Through Train” Project (No. CSTB2022BSXM-JCX0022), and Chongqing Shapingba District 2024 Techmology lnnovation Project (No. 2024114).

## Conflict of interests

The authors declared no competing interests.
